# The Napping Behaviour of Australian University Students

**DOI:** 10.1371/journal.pone.0113666

**Published:** 2014-11-20

**Authors:** Nicole Lovato, Leon Lack, Helen Wright

**Affiliations:** School of Psychology, Flinders University, Adelaide, SA, Australia; Oasi research institute, Italy

## Abstract

The purpose of this study was to evaluate the self-reported sleep and napping behaviour of Australian university students and the relationship between napping and daytime functioning. A sample of 280 university first-year psychology students (median age  = 19.00 years) completed a 6-item napping behaviour questionnaire, a 12-item Daytime Feelings and Functioning Scale, the Pittsburg Sleep Quality Index and the Epworth Sleepiness Scale. Results indicated that 53.6% of students reported napping with 34% napping at least 1–2 times per week, and 17% napping three or more occasions per week. Long naps, those over 30 minutes, were taken by 77% of the napping students. Sixty-one percent of students reported they took long naps during the post-lunch dip period, from 2–4pm. Students who nap at least once per week reported significantly more problems organizing their thoughts, gaining motivation, concentrating, and finishing tasks than students who did not nap. Students who napped also felt significantly more sleepy and depressed when compared to students who did not nap. The results also indicated that nap frequency increased with daytime sleepiness. The majority of students (51%) reported sleeping 6–7 hours per night or less. Overall, the results from this study suggest that among this population of Australian first-year university students habitual napping is common and may be used in an attempt to compensate for the detrimental effects of excessive sleepiness.

## Introduction

Experimental research has consistently demonstrated that napping during the day improves objective and subjective alertness, cognitive functioning, psycho-motor performance, memory and even mood [1]–[7]. Although these effects are well established it remains relatively unknown whether routine napping is common in populations who are likely to benefit from improvements in alertness. Research shows that, for example 25–50% of university students report significant levels of daytime sleepiness [8]–[10]. The increase in electronic media use in the evening and at bedtime during adolescence and young adulthood has been consistently demonstrated to contribute to heightened sleepiness during the day by delaying bedtimes and shortening the nocturnal total sleep time of this population [11]–[13]. Excessive daytime sleepiness has a detrimental influence on cognitive functioning and psychomotor performance [14]–[16] and therefore can not only impact upon academic performance but also on tasks such as driving, adding to the risk for motor vehicle accidents in these relatively inexperienced drivers.

Several research groups have recognized the high prevalence of excessive daytime sleepiness in this population and have consequently investigated their sleep patterns. Survey studies have reported university students typically sleep for 7–8 hours per night, with average bedtimes ranging from midnight to as late as 01∶30 h [10], [14], [16], [17]. However, very little research has investigated whether students engage in behaviours such as napping to alleviate sleepiness.

To the authors' knowledge, only two studies have reported the napping behaviour of university students. Tsai and Li surveyed 237 Taiwanese students with a mean age of 20.74 years [16]. These students reported a mean nocturnal sleep duration of 7 hours and 20 minutes, and a median nap length of approximately 27 minutes. Females reported taking significantly longer naps than males. Tsai and Li also investigated the relationship between nap length and academic performance [16]. Despite longer naps being related to poor sleep quality and increased awakenings at night, nap length was not significantly associated with academic performance.

In a similar study, Bahammam, Al-Khairy, and Al-Taweel [18] surveyed the sleep of 129 medical students in Saudi Arabia (mean age  = 21 years) and reported a mean nocturnal sleep duration of 5 hours and 54 minutes. Eighty-three percent of the students sampled reported napping with a mean duration of 1 hour and 15 minutes. Unfortunately, the number of naps taken per week and the timing of these naps throughout the day was not reported in this study or that of Tsai and Lee [16].

The current study investigated the prevalence of napping, number of naps, typical timing of naps and the reasons for napping in Australian university students. The secondary aim was to assess the relationship between napping and daytime functioning reported by students, a relationship which has been scarcely addressed in the literature.

## Method

### Participants

**Figure 1:**
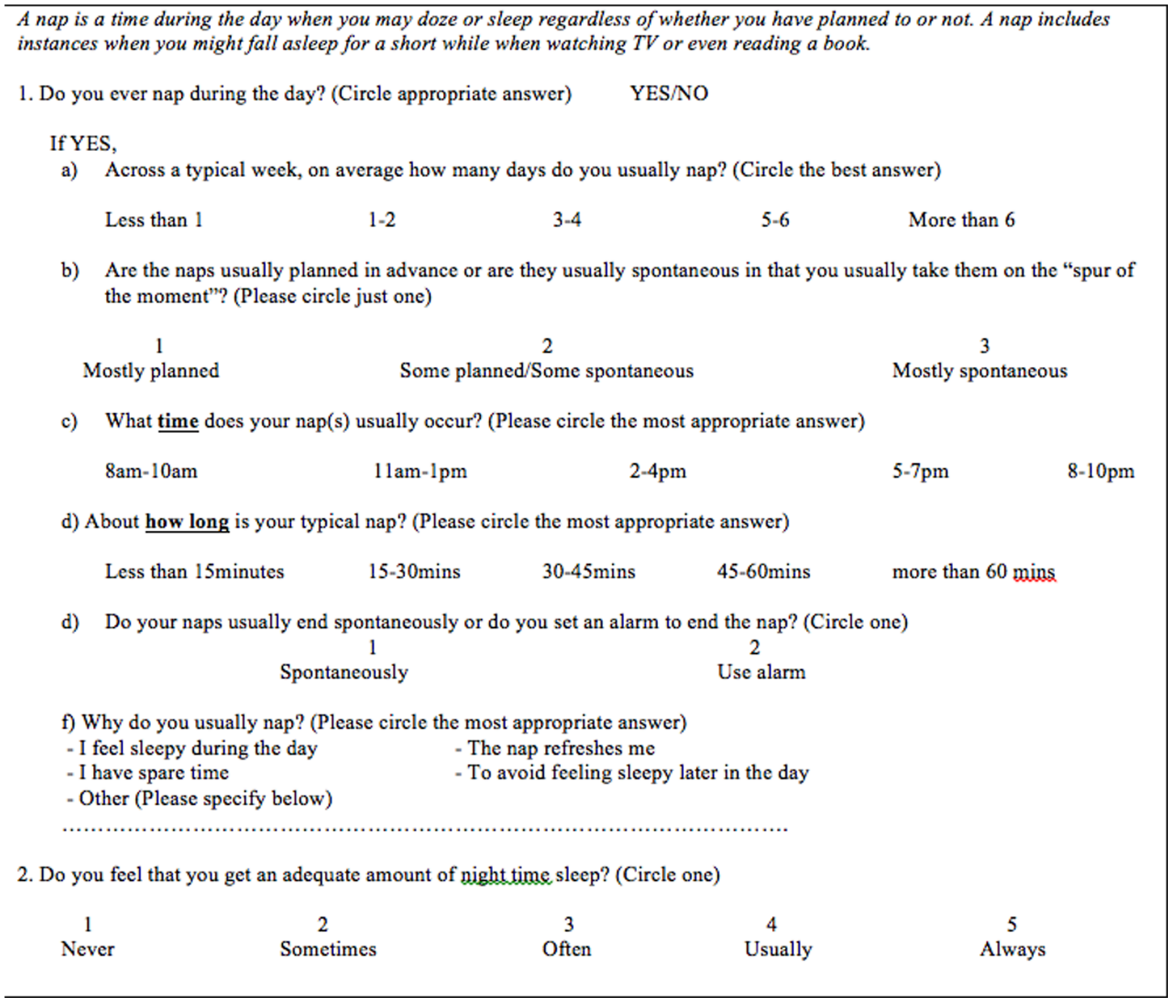
Napping Behaviour Questionnaire.

The sample included 280 (52 male, 228 female) university first-year psychology students aged 16–60 years (*Median* = 19.00, *SD* = 7.73). Participants were required to complete a 6-item napping behaviour questionnaire (see Figure 1) that asks students if they nap and includes items regarding the characteristics of naps such as frequency, duration, time of day when any naps are taken and the reason for napping. Participants were also required to complete the Pittsburgh Sleep Quality Index (PSQI) [19], the Epworth Sleepiness Scale (ESS) [20], and the Daytime Functioning and Feeling Questionnaire (DFFS) [21].

The study received approval from the Flinders University Social and Behavioural Research Ethics Committee. All participants provided their written consent to participate in this study. Written informed consent was obtained from either the next of kin, caretaker, or guardians on behalf of the minors enrolled in this study.

## Results

Just over half of the students sampled (54.6%) reported napping at least 1–2 times per week. For the purpose of further analysis, these students were classified as nappers. When the nappers were asked how often they napped, 68% reported napping one to two occasions per week, while 32% reported napping three or more times per week.

The majority of naps occurred spontaneously during the “post-lunch dip” period, with 61% of the napping students reporting they typically took naps between 2–4pm. Long naps, those over 30 minutes, were taken by 77% of the nappers.

The student sample as a whole reported going to bed at 22∶50 h and the majority reported sleeping for only 6–7 hours or less. Bedtime did not differ significantly between the napping and non-napping students (*p* = .767), nor did total sleep time (*p* = .143). As shown in Table 1, only 3.2% of students reported their nocturnal total sleep time was always adequate. Forty-nine percent of students reported their amount of sleep at night as adequate only some of the time. Chi-square analyses revealed no significant differences between the nappers and non-nappers in their reports of obtaining an adequate amount of nocturnal sleep (all *ps*>.05).

**Table 1:** Distribution (proportion) of estimated sleep need for nappers and non-nappers.

	Never	Sometimes	Often	Usually	Always
Overall	5.0	48.9	18.7	24.1	3.2
Nappers	6.1	48.1	19.3	23.6	2.8
Non-nappers	1.5	51.5	16.7	25.8	4.5

Excessive daytime sleepiness was the most commonly reported reason for napping. These feelings were reflected by significantly higher daytime sleepiness scores (ESS) for students who napped (*M* = 8.26, *SD* = 3.34), when compared to students who did not nap (*M* = 5.58, *SD* = 3.07), *p*<.001. Daytime sleepiness was positively correlated with nap frequency (*r* = .503), *p*<.001. Students who reported napping three or more times per week had mean daytime sleepiness scores ranging from 10–12 (*M* = 10.33, *SD* = 3.34), indicating excessive levels of daytime sleepiness.

Separate t-tests were conducted to assess the daytime functioning and feelings of napping and non-napping students, see Table 2.

**Table 2:** T-tests for the total DFFS score and the individual items for napping and non-napping students.

	Nappers (*N* = 153)	Non-nappers (*N* = 127)	*P (2-T)*
Overall DFFS score	12.80 (6.62)	11.00 (6.15)	.021 *
Felt lethargic	1.44 (.77)	1.30 (.78)	.150
Felt irritable	1.14 (.69)	1.08 (.76)	.470
Lacked motivation	1.54 (.85)	1.32 (.81)	.028 *
Been unable to concentrate	1.45 (.72)	1.26 (.70)	.209 *
Had trouble with poor memory	.93 (.87)	.93 (.85)	.970
Felt fatigued	1.52 (.76)	1.45 (.84)	.443
Had difficulty accomplishing daytime tasks	.92 (.86)	.74 (.73)	.046 *
Found it difficult to enjoy social interaction	.64 (.83)	.60 (.68)	.653
Felt ill	.68 (.85)	.55 (.81)	.201
Felt you had a reduced quality of life	.69 (.84)	.52 (.79)	.090
Found it difficult to organize your thoughts	1.03 (.84)	.76 (.82)	.007 *
Felt depressed	.81 (.92)	.50 (.70)	.002 *

Note: DFFS refers to the Daytime Feeling and Functioning Scale.

*denotes significant differences between napping and non-napping students.

The napping students reported significantly worse overall daytime functioning and feelings when compared to the students who did not nap. In particular napping students reported significantly more problems organizing their thoughts, gaining motivation, concentrating, and finishing tasks than students who did not nap. Student who napped also reported more feelings of depressed mood than the students who did not nap.

## Discussion

The present study assessed the self-reported sleep and napping behaviour of a sample of 280 Australian university students. A minority of the students in this sample reported sleeping for over 7 hours per night with an average bedtime of 22∶50 h. Fifty-four percent of the students in this sample reported napping on at least 1–2 occasions per week, most napping two or more times per week. The naps were primarily over 30 minutes in duration and occurred spontaneously during the post-lunch dip period. When students were asked why they took naps, a feeling of daytime sleepiness was cited as the most common reason. This feeling of daytime sleepiness was reflected in scores on the Epworth Sleepiness Scale, which indicated those students who napped were significantly more sleepy than those who did not and students who napped on three or more occasions per week reported daytime sleepiness scores within the excessive range.

Given nocturnal subjective total sleep time did not differ between the napping and non-napping students, the source of the discrepancy in excessive daytime sleepiness between the two groups of students is unclear. One potential explanation however lies with the method used to measure total sleep time. Participants were required to indicate their estimated total sleep time by selecting one of four categorical options, either 0 = >7 hours, 1 = 6–7 hours, 2 = 5–6 hours, or 3 = <5 hours. Forty-nine percent of students indicated their estimated total sleep time was over 7 hours, therefore the lack of precision at this end of the sleep length distribution may make statistical comparisons unreliable. Future research should employ a more fine-grained measure of total sleep time and other sleep quality variables to further investigate the underlying cause of excessive daytime sleepiness in the napping students.

Alternatively, the nappers as a group may have higher than average sleep need. In this case, obtaining the same amount of total sleep as the non-nappers would result in greater relative sleep loss and accumulation of homeostatic sleep drive and sleepiness.

The prevalence of napping among the current sample of students was less than found by Bahammam and colleagues [18], who reported 83% of their student sample napped on two or more occasions during the week. The average nap length reported by Bahammam and colleagues was 1 hour and 15 minutes, which is much longer than the length reported by students in the current sample [18]. Only 15% of the students sampled in the current study reported taking naps lasting longer than 1 hour. It is important to note, however that the nocturnal sleep duration reported by students in the study by Bahammam and colleagues was nearly two hours less than the duration reported by the sample in the current study [18]. Additionally, Saudi Arabia adopts a siesta culture whereby these students had a greater opportunity to nap than those in the current study. These factors therefore are likely to explain the elevated prevalence of napping and longer nap duration in these students relative to the current study.

The current study also investigated the relationship between napping and daytime functioning reported by students, a relationship which has not received adequate attention in the past. Students who took naps reported significantly worse overall daytime functioning when compared to the non-napping students. Students who napped reported significantly more problems organizing their thoughts, gaining motivation, concentrating, and finishing tasks than students who did not nap. Napping students also reported greater feelings of depressed mood when compared to non-napping students. Because experimental studies generally show benefits of napping to all these outcome variables, the association between napping and poor daytime functioning in the present study is unlikely to arise from a negative effect of the napping. More likely, these results suggest that students may use (voluntarily or spontaneously) naps to compensate for excessive sleepiness arising from insufficient amount of sleep.

The subjective reports of daytime feeling and functioning in the current study were in response to questions asking about levels of functioning over the previous 2 weeks and most likely to relate to the participants chronic daytime feelings. Therefore these answers may not reflect any acute and relatively short lived potential improvements in daytime feeling and functioning following a nap. Although past research has demonstrated naps improve alertness and functioning following a nap, this effect has been established under strict laboratory conditions with individuals who do not regularly nap. Future research would benefit from assessing subjective and objective functioning in individuals who nap on a regular basis in response to sleepiness feelings rather than at experimentally set times.

It is important to note the current sample is relatively small and comprises primarily female psychology students in their first year of study. Although there were no gender differences in any sleep, napping or daytime functioning variable examined, future research should endeavor to seek a broader, larger sample of students. Future research is also encouraged to assess nocturnal sleep, napping behavior, and daytime functioning objectively to confirm the current subjective reports.

Overall, the results from the current study suggest habitual napping is common among this population of primarily female Australian first-year university students. Napping in this sample may be used to compensate for the detrimental effects of excessive sleepiness on daytime functioning.
